# Editorial: Cervical screening awareness week 2023: integrating cervical cancer screening and precancer treatments

**DOI:** 10.3389/fonc.2025.1614832

**Published:** 2025-05-21

**Authors:** Noleb M. Mugisha, Leeya F. Pinder, Manoj P. Menon

**Affiliations:** ^1^ Comprehensive Community Cancer Program, Uganda Cancer Institute (UCI), Kampala, Uganda; ^2^ College of Medicine, University of Cincinnati, Cincinnati, OH, United States; ^3^ Vaccine and Infectious Disease Division, Fred Hutchinson Cancer Center, Seattle, WA, United States

**Keywords:** cervical cancer, screening, vaccine, cost effectiveness, barriers and facilitative factors

## Introduction

Despite effective methods to both prevent and screen for cervical cancer, invasive cervical cancer (ICC) remains a leading cause of morbidity and mortality globally. While the incidence of ICC and mortality secondary to ICC has declined dramatically in high-income countries (HICs) – these gains are not universal. Indeed, ICC is the most common cause of cancer-related death in many low-and-middle-income countries (LMICs). As such, there is an urgent need to develop and implement locally relevant interventions to achieve the World Health Organization (WHO) 90-70-90 cervical cancer elimination targets. The WHO aims to vaccinate 90% of girls, screen 70% of women, and treat 90% of women with cervical disease by 2030, however novel efforts are necessary to achieve these ambitious goals (1). Here, we highlight strategies which target cervical cancer awareness, screening, prevention, and treatment – in an economically sustainably fashion.

## Awareness

Given the relatively low rates of cervical cancer screening in Ethiopia, Yosef et al. via a hospital-based case-control study demonstrated that knowledge of cervical cancer screening and proximity to the health facility were associated with cervical cancer screening. Recognizing the vital need of cancer awareness, Ouedraogo et al. organized a workshop, consisting of representatives from the relevant stakeholders- including government, non-governmental organizations, civil society organizations, and academic/research organizations, to craft and tailor effective health education and communication strategies.

## Screening

To assess the availability and capacity of cervical cancer screening and treatment services in Kenya, Mwenda et al. conducted a sub-national survey of healthcare workers in over 3,000 hospitals. Only 5% of hospitals provided both cervical cancer screening and treatment services – a disparity which will need to be addressed in order to achieve the WHO targets.

## Prevention

The prevention of cervical cancer incorporates both primary prevention strategies, via an effective vaccine, as well as secondary prevention, via treatment of pre-cancerous lesions. To inform the implementation of the most effective vaccine, Kebede et al. describe the prevalence and variation of HPV genotypes. Given that prevalence of genotypes which are not included in the commonly used bivalent or quadrivalent HPV vaccine, such information can help advocate for nonavalent vaccine.

Hypothesizing that with increased utilization of the HPV vaccines, the genotypes of HPV may vary among those vaccinated and those unvaccinated Yang et al. conducted a study among women with atypical squamous cells of undetermined significance (ASCUS). These researchers concluded that genotype identification may inform the choice of triage options for women identified to have ASCUS lessions on cervical cytology screening. Although the goal to increase HPV remains, cost-effective triage strategies are particularly necessary in resource-limited regions.

Lee et al. explored the feasibility and acceptability among women undergoing various HPV-based screen-triage-treatment options, including self-collected vaginal samples. These researchers offer specific strategies, including the need for health education to optimize perspectives and utilization of cervical cancer prevention services. To minimize the invasive procedures, Qian et al. investigated the safety and efficacy of the non-invasive 5-aminolevulinic acid photodynamic therapy (ALA-PDT) in the treatment of high-grade squamous intraepithelial lesions. Although ALA-PDT has been used in low-grade squamous intraepithelial lesions, these researchers found that the treatment was safe – with no severe adverse effects, as well as effective – with a 12-month complete regression rate of over 80%.

Mungo et al. conducted focus group discussions to learn of men’s perspectives on their female partner’s use of topical therapies for pre-cancerous lesions. These colleagues also encourage health education strategies which reach the partners of patients undergoing cervical precancer treatment.

Given the need for cost-effective strategies for the diagnosis and treatment of cervical cancer and pre-cancerous lesions and recognizing the pathogenesis of cervical cancer, Gong et al. developed a Classification of Lesion Stages (CLS) algorithm to predict the risk of cervical cancer. The use of such technology may optimally triage patients with pre-cancer lesions, reduce the number of unnecessary procedures, and potentially alleviate the burden on health systems.

Although the screening modalities vary among resource-abundant and resource-limited regions, the benefits of resource-relevant screening are clear. An obvious challenge of cervical cancer screening programs is the limited funds dedicated to cancer prevention. Tran et al. utilized a simulation model of the current standard of care (i.e. cytology and colposcopy triage) with various scenarios calculated the disability-adjusted life-years (DALYs) averted for each scenario. These researchers demonstrated that repeat HPV DNA testing was associated with the highest DALY averted.

Clinical services in LMICs are often funded and provided in a vertical fashion with the appropriate integration of relevant infrastructure (2, 3). Because many health systems do not have a primary care model of service delivery, there has been increased recognition of the need to leverage and incorporate non-communicable disease (NCDs) care, including cancer care, within the existing routine services. The benefit of such integration has been demonstrated for certain NCDs, including hypertension and diabetes (4, 5). However, despite the clinical burden, such an integrated approach has not been fully implemented for the early detection of cervical cancer and precancer care treatment.

The articles in this Research Topic highlight the burden of cervical cancer as well as necessary strategies to decrease the toll which disproportionately impacts LMICs. Although effective vaccines to prevent cervical cancer and screening techniques to identify pre-cancerous lesions exist, disparities persist with regard to clinical access. As such, the unnecessary burden cervical cancer remain. Further cost-effective efforts to incorporate the findings demonstrated in this Research Topic and specifically integrate cervical cancer screening and precancer treatment programs within existing health care programs are necessary to achieve the WHO cervical cancer elimination targets.
